# Size fractionation of high-density polyethylene breakdown nanoplastics reveals different toxic response in *Daphnia magna*

**DOI:** 10.1038/s41598-022-06991-1

**Published:** 2022-02-24

**Authors:** Mikael T. Ekvall, Isabella Gimskog, Jing Hua, Egle Kelpsiene, Martin Lundqvist, Tommy Cedervall

**Affiliations:** 1 Aquatic Ecology Unit, Department of Biology, Ecology Building, Lund University, 223 62 Lund, Sweden; 2 NanoLund, Lund University, Box 118, 221 00 Lund, Sweden; 3 Biochemistry and Structural Biology, Lund University, Box 124, 221 00 Lund, Sweden

**Keywords:** Environmental sciences, Materials science

## Abstract

Plastic litter is a growing environmental problem. Recently, microplastics and nanoplastics, produced during breakdown processes in nature, have been in focus. Although there is a growing knowledge concerning microplastic, little is still known about the effect of nanoplastics. We have showed that mechanical breakdown of high-density polyethylene (HDPE), followed by filtration through 0.8 µm filters, produces material toxic to the freshwater zooplankton *Daphnia magna* and affected the reproduction in life-time tests. However, further size fractionation and purification reveals that the nanoplastics fraction is non-toxic at these concentrations, whereas the fraction with smaller sizes, below ~ 3 nm, is toxic. The HDPE nanoplastics are highly oxidized and with an average diameter of 110 nm. We conclude that mechanical breakdown of HDPE may cause environmental problems, but that the fraction of leached additives and short chain HDPE are more problematic than HDPE nanoplastics.

## Introduction

Plastic pollution is a commonly recognized environmental problem. The mass of produced plastics is increasing and 368 million tons were produced worldwide in 2019^1^. With the increasing production, the plastics ending up in the environment is likewise increasing. The mismanaged plastic waste was estimated to be between 60 and 99 million tons with a majority of it, 91%, ending up in waterways and potentially reaching the oceans^2^. The most commonly produced plastics in Europe are in falling order: Polypropylene (PP), Low-density polyethylene (LDPE), High-density polyethylene (HDPE), Polyvinyl chloride (PVC), polyurethane (PUR), polyethylene terephthalate (PET), and polystyrene (PS)^1^. In the aquatic environment PE is dominating the waste followed by PP and PS^3^.

Nanoplastics have been suggested to be defined as plastic broken down in nature, opposite to engineered polymeric nanoparticles^4^, with an upper size of either 0.1 or 1 µm depending on the used definition. The definition of nanoplastics and their unique features has recently been discussed^5^. It is worth mentioning that the definition of nanoparticles, including plastic materials, is that at least one dimension of the particle is below 100 nm. Nanoplastics have been collected in the North Atlantic Gyre^6^. However, nanoplastics in larger quantities are difficult to collect and identify in natural samples due to their small size and carbon-based chemistry^7^. Moreover, there is not a generic nanoplastic sampling method that can be applied to different types of environmental samples.

Reference material of small (below 200 nm) nanoplastics are only available for a few plastics e.g. PS, Poly(methyl methacrylate), and poly(lactic-co-glycolic acid). Of these the most used in environmental studies and the only environmental relevant plastic is PS. The results of these studies have been extensively reviewed before^8–11^ and have for example taught us that small size and positive charge of the modified surface has more adverse effects than larger size and negative charge. However, the reference material is usually in the form of perfect spheres and often with a modified surface chemistry that may not represent natural breakdown nanoplastics. Therefore, in order truly evaluate the effect of nanoplastics in nature we need to use more environmentally realistic nanoplastics and from all the relevant sorts of plastics.

The current lack of NPs collected from the environment, and hence environmentally relevant reference NPs, have recently led to several attempts to induce the release of nanoplastics from plastics or artificially break down plastics to nano size. It has been shown that nano-sized particles can be produced after PS cup lids being exposed to ultraviolet (UV) radiation^12^. Similarly, PS cup lids and expanded PS have mechanically been broken down to an average size of around 140 nm using a food processor^13^. Treating nylon tea bags with hot water resulted in the release of both micro- and nano-sized plastic particles^14^. PS particles with a diameter of 200 nm were degraded to NPs below 100 nm by agitation in water^15^. Different sources of shear forces have been shown to break down PE microplastic originating from facial scrub to nanosized particles with an average size between 51 and 73 nm^16^. PET have been broken down by laser ablation to an average size of 100 nm^17^ and mechanically^18^ by a similar method as for PS and expanded PS^13^. Thermal oxidation and soft mechanical forces on expanded PS resulted in nanosized particles^19^. Nanosized particles have also been observed in leachate from a mix of plastics after subjected to sunlight^20^. Despite, these different experimental approaches to produce nanoplastics there is so far no general method to produce nanoplastics from different sources of plastics.

When particles with sizes within the nanoplastics size range, i.e., below 1 µm, are identified, the isolation and chemical characterization of the nanoplastics are often lacking, leaving the nature of the breakdown nanoplastics unconfirmed. In a few cases the breakdown nanoplastic is characterized for chemistry, surface chemistry, and/or shape^13,14,17^. In general, the leached and broken down nanoplastics are different from the reference PS nanoplastics. The size distribution is broader, changed surface chemistry and multiple structures including sharp edges. Therefore, breakdown nanoplastics should be a more environmentally relevant test material of nanoplastic effects than the PS reference material.

Microplastic particles (< 5 mm), similarly as nano-sized plastics, can be generated from the fragmentation of larger plastic pieces or enter the environment directly as microscopic fragments^21^. In contrast to nanoplastics, microplastics are available from many different plastics and it is easier to break down plastics to microplastics^21^. As a result, toxicity data can be obtained for different plastics including PE. Microplastics, including PE, have been extensively studied in several aquatic organisms, such as mussels, lugworms, crustaceans such as *D. magna*^22^. In acute exposure scenarios PE microplastics have size-dependent negative effect on *D. magna* survival^23–25^, whereas in the presence of algae no effect was seen after in 21 days exposure of PE microplastics^26^. Interestingly, irregular PE microplastics were more acutely toxic than spherical particles^25^ which emphasizes the need to use irregularly shaped nanoplastics in addition to the spherical PS model particles.

However, there are few studies reporting on how leached or broken down nanoplastics exhibit biological effects, for example, in cell lines^17^, *D. magna*^14,20^, and on zebrafish (*Danio rerio*)^18^, however, there is an acute need for more data in order to draw any conclusions. Nonetheless, there are several methodological challenges and pitfalls studying nanoplastics. It has recently been noted that effect studies on PS reference material are influenced by additives such as Tween^®^, that stabilizes the particle dispersion, and sodium azide, that prohibit bacterial growth^27,28^. In the process of breaking down plastics to nanoplastics it is reasonable to expect increased leaching of additives that can affect the test organisms. This has been noted in for example the material leached from tea bags as removal of small molecules by dialysis decreased the biological effect of the remaining micro- and nanoplastics^14^. Therefore, to evaluate the effect of breakdown nanoplastics or different size fractions of microplastics the particles need to be isolated from both smaller and larger molecules and particles.

In the present study, we have mechanically broken-down HDPE bags and have removed macro- and microplastics. The remaining material were separated into a nanoplastic fraction and a fraction consisting of smaller (< 3 nm) molecules, which then were tested for toxicity using the freshwater zooplankton *D. magna*.

## Materials and methods

### Preparation of breakdown HDPE nanoplastics

#### For the 1st toxicity test on *D. magna*

Plastic nanoparticles were prepared from HDPE packaging material (Fill-Air Extreme from SealedAir Product care). The material was chosen to be representative of a plastic product that can be present as plastic pollution in marine environments. The PE plastic was broken down using a similar method previously applied to PS^13^. Two grams of plastic were cut up in small pieces (approximately 1 cm^2^). Only the plastic without print was used to eliminate the potential effects of the pigments. The plastic pieces were put in a glass beaker with 200 mL of tap water (detailed information about the water can be found in Table S1). The plastic was then fragmentized in the water using a Bosch ErgoMixx 600 W hand-held food blender at the highest speed setting for two minutes. After the blending, 100 mL was filtrated using a 0.8 µm syringe filter and placed into a separate glass bottle. To the remaining plastic and water mix another 100 mL of tap water was added and again fragmentized by the food blender. This procedure was repeated until 500 mL of filtrated “plastic water” was attained. This sample is called “Polyethylene breakdown” (PEBD) and contains all molecules and material that can make it through a 0.8 µm syringe filter.

The PEBD was further fractionated by filtration and concentration using a VivaFlow device (VIVAFLOW 50R, HY, Sartorius) with a 10 kDa filter and ran at a flow rate of approximately 250 mL/min. The total volume of the PEBD sample was concentrated to a fifth of the original (i.e. 100 mL), this fraction is referred to as “Polyethylene VivaFlow” (PEVF) and contains molecules that are larger than ~ 10 kDa (for a HDPE particle that would correspond to a size of approximately 3 nm in diameter). Before the toxicity test the sample was diluted 5 times in tap water.

The collected filtrate is referred to as “FT”. We hypothesize that the particles will be concentrated in the PEVF and only the smallest particles, chains of PE that has been broken from the particles, and potential solved additives will go through the VivaFlow device into the FT fraction.

These samples: PEBD, PEVF and FT were used together with a control sample (tap water) in the 1st toxicity test on *D. magna* (see below).

The PEVF fraction was further fractionated for size characterization by filtration through 450 and 200 nm spin filters, (Spin-X, Corning Incorporated, USA), and 100 nm spin filters, (Ultrafree-MC Centrifugal Filters, Merck Millipore).

#### For the 2nd toxicity test on *D. magna*

**Figure 1:**
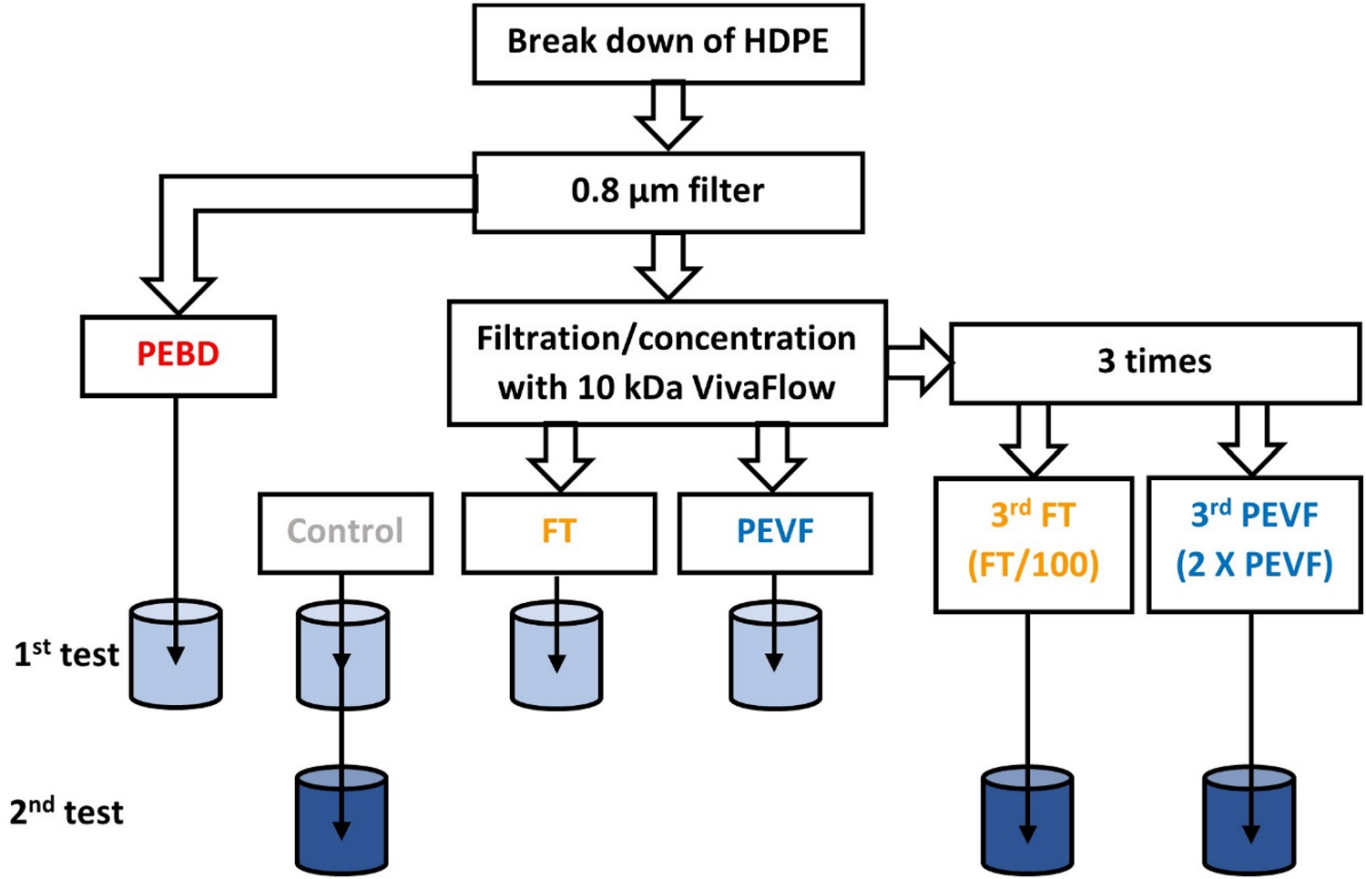
Scheme of HDPE break down process. Two grams of HDPE is mechanically broken down in water using a food blender. The water is and filtrated through a 0.8 um filter. In the 1st test the nanoplastics are concentrated and separated from smaller molecules using a VivaFlow device with a molecular weight cut off 10 kDa and the toxicity of each fraction and control was tested on *D. magna*. In the 2nd test the nanoparticles are purified from smaller molecules through repeated (three times) concentration and dilutions. At the end the PEVF is concentrated two times and the smaller molecules in PEVF and FT less than 1% compared to in 1st toxicity test.

The procedure outlined above was followed. However, with only one concentration step the PEVF sample used in test 1 contains ~ 20% of FT (the smallest particles, chains of PE that has been broken from the particles, and potential solved additives). To reduce the concentration of FT in the PEVF fraction the concentration step was repeated by diluting the water and then concentrated again with the VivaFlow device. This process was repeated three times resulting in the samples 3rd PEVF and 3rd FT, which were used together with a control sample (tap water) in the 2nd toxicity test on *D. magna* (see below). These samples, except the control, contains ~ 1% of the smallest particles, chains of PE that has been broken from the particles, and potential solved additives compared to the concentration of these in the PEVF sample in the 1st test. A schematic view of the experimental set up is outlined in Fig. 1.

### Characterization of breakdown HDPE nanoplastics

The size and number concentration of the nanoplastics in the different PE nanoplastics fractions were investigated by nanoparticle tracking analyses (NTA) using NanoSight LM10 (Amesbury, UK). The concentrated fraction (PEVF) was diluted with MilliQ-water to half the concentration to obtain good measurements. The obtained recordings, at camera level 14 for 30 s, were analysed with software NanoSight NTA 3.1 with a standard analysis setting. All reported data is an average of 3 to 5 separate recordings. The average particle size was also determined by dynamic light scattering (DLS) using a DynaPro Plate Reader II, Wyatt Technology Corp, USA. The dynamic light scattering was, for each sample, recorded at 23 °C for 10 s 10 times, and the obtained data was analysed using the Dynamics V7 program. Zeta potential measurements were performed in tap water at 25 °C using a Zetasizer Nano ZS instrument (Malvern Instruments, Worcestershire, UK). Measurements were repeated three times and averaged for three consecutive analyses of the same sample. UV absorbance spectra were obtained from all fractions in a 1 cm quarts cuvette between 200 and 350 nm using a ProbeDrum spectrophotometer (Probation Labs, Sweden). The pH of the fractions was measured on a Seven Compact (Mettler Toledo) pH meter for all fractions before and after the addition of algae. The pH was around 7.8 for all breakdown fractions and around 8.0 after algae was added.

Transmission electronic microscopy (TEM) images were obtained on a JEOL JEM-1400 PLUS microscope at 100 kV (JEOL Ltd., Japan) using TEM Centre for JEM1400 Plus software. The samples were prepared by placing 10 μL of sample onto a pioloform-coated single slot grid (Ted Pella, Cu, Pelco Slot Grids, USA), using a pipette. The samples were set aside to let water evaporate for a couple of days before analysis.

Attenuated total reflectance Fourier transformed infrared spectrometry (ATR-FTIR) was performed on a Spectrum One FT-IR spectrometer (Perkin Elmer) equipped with a Universal ATR accessory. The samples (5 μL) were added to the crystal and evaporated. The spectra were obtained using software Spectrum version 6.2.0 and a Spectral resolution of 4 cm^−1^ in the range of 4000–550 nm.

### Toxicity test 1 on *D. magna*: high concentration of flow through molecules

The test fractions used for the long-term toxicity testing on *D. magna* were prepared in the same way as the fractions used to characterize the particle containing fractions and establish the reproducibility of the process (see above), but in a larger volume (1.4 L for each fraction). Before the start of the test the PEVF fraction was diluted to its original volume.

From each fraction 70 mL was distributed into 20 uncovered glass beakers. A control was made from only tap water. To each beaker one *D. magna* was added and 10 mL of green algae (*Scenedesmus* sp.), rendering the total volume in each jar to be 80 mL. This resulted in a final algae concentration of 450 μg/L (measured using an AlgaeLabAnalyser, bbe Moldaenke, GmbH). All added *D. magna* were neonates (less than 24 h old) when the experiment started. The beakers were marked and randomly placed on a table under fluorescent lamps. Animals that died within the first 48 h were replaced and their deaths not included in the statistics. This was performed to not include deaths from possible injuries attained in the pipette transfer of organisms from the culture aquaria to the test vessels. The test animals were maintained at a temperature of 18 °C at a 12:12 h light/dark photoperiod.

The animals were fed once per week with 5 mL of green algae and the evaporated water was replaced at the same frequency (approximately 5 mL per week). The beakers were checked every weekday. Immobilization and offspring were recorded, and the offspring were counted and removed using a Pasteur pipette as soon as detected. The experiment proceeded until all *D. magna* were immobilized, here 134 days.

### Toxicity test 2 on *D. magna*: high concentration of PEVF and low concentration of FT

A second long-term experiment was performed as a complement to the first one, focusing on the concentrated and re-diluted fraction of nanoplastics here referred to as PEVF. This procedure was similar to the first experiment, but the particles were “cleaned” (see details above) three times using the VivaFlow device and then re-diluted to half its original volume. The particles were concentrated ten times, then re-diluted and this process was repeated three times. This procedure was done to eliminate the smallest particles. At the final re-dilution the particles were only diluted to half their original volume. This cleaned fraction is referred to as 3rd PEVF. The FT fraction in this test was attained at the third and final concentration step and should therefore only contain ~ 1% of the original molecules under 10 kDa, we refer to this fraction as 3rd FT. Another difference between the tests were that the particle solution was changed every week to keep the exposure higher and more controlled and the total volume of water in the beakers were 40 mL instead of 80 mL as in the first experiment, the algal concentration was kept at the same level as in test 1. The experiment proceeded until all *D. magna* were immobilized, here 98 days.

### Statistical analysis

Statistical analysis of the results was performed using statistical computing software GraphPad Prism version 8.0.0 (224) for Windows (GraphPad Software, Inc., www.graphpad.com). The analysis of the survival was performed with the (Log-rank) Mantel-Cox test as well as the Gehan-Breslow-Wilcox test. The reproduction was analysed as number of offspring per animal and day lived using a one-way ANOVA and Tukey’s multiple comparison test that compares the mean of every group.

## Results and discussion

### Characterization of the broken-down plastic

**Figure 2:**
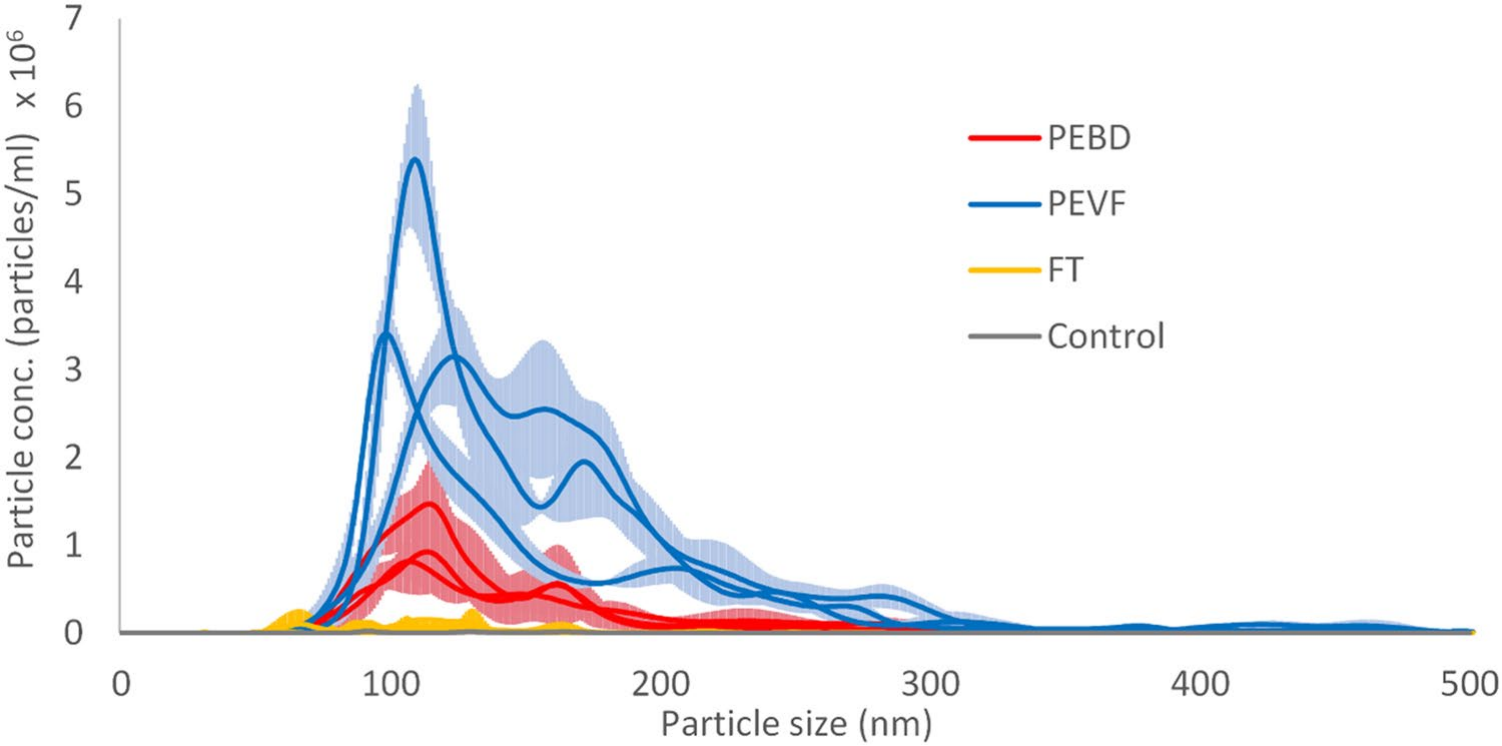
The size distribution of HDPE breakdown fractions determined by NTA. Each sample is an average from five recordings. The graphs represent the mean of these recordings and the lighter area around the graph represents the standard deviation from the runs. A control sample of the tap water is shown in grey line.

The breakdown procedure of the PE plastic bag was repeated on three separate samples and resulting fractions analysed separately to evaluate the reproducibility of the process. NTA measurements of PEBD and PEVF showed a mean particle concentration of the breakdown nanoplastics of 7.5 × 10^8^ and 2.9 × 10^9^ particles per mL, respectively (Table 1), i.e., the particle concentration in PEVF is about 4 times higher which is expected as the PEVF is concentrated 5 times in the breakdown process. The peak sizes of PEBD and PEVF fractions are between approximately 90 and 200 nm (Fig. 2, Table 1), although, small populations of nanoplastics have larger sizes. From the average concentration and size, the mass concentration was calculated (Table 1). However, there is a sharp decrease in the NTA size distribution data below 90 nm. This may be due to that there are no smaller nanoplastics in the sample, but could, alternatively, be due to the NTA detection limit, which for polystyrene nanoplastics is around 60–80 nm. Consequently, we cannot from the NTA measurements rule out that there is a population of smaller nanoplastics. The x-axis has been cut off at 500 nm as no populations were found between 500 and 1000 nm even though the sample had been passed through a 0.8 μm syringe filter. Hence the breakdown method generates nanoparticles with a narrow size range from the macro-HDPE sample.

**Table 1:** NTA data for the different fractions.

Sample	Peak concentration (particles/mL)		Peak diameter (nm)		Mass concentration^b^ (µg/mL)
	Individual sample^a^	Average	Individual sample	Average	Average
PEBD 1	6.72e^+8^		114		
PEBD 2	6.92 e^+8^	7.5e^+8^	108	112	0.53
PEBD 3	8.86 e^+8^		115		
PEVF 1	2.18e^+9^		98		
PEVF 2	3.18e^+9^	2.9e^+9^	125	111	2.0
PEVF 3	3.28e^+9^		110		

^a^The value is the average of five individual measurement of the specific sample.

^b^The value is calculated using the density 0.97 g/mL.

**Figure 3:**
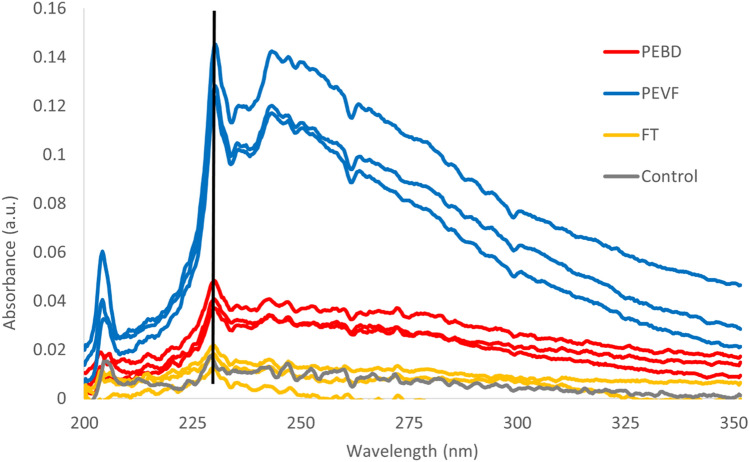
Absorption spectra of HDPE breakdown fractions. Absorbance measurements for the triplicate break down fractions and a tap water used as a control. Black vertical line highlights peak maxima at 230 nm.

Absorbance was measured for all PEBD breakdown fractions (Fig. 3). The absorbance spectra have maxima and minima at similar wavelengths. However, the ratios between different peaks are different. Compare for example maxima at 230 and 205 nm from PEVF and PEBD and FT. The absorbance peak at 230 nm is about 4 times stronger for PEVF compared with PEBD confirming that the concentration of PE is higher.

The morphology of the breakdown PE nanoplastics was characterized by TEM. The formed HDPE nanoplastics were irregular in both size and shape (Fig. S1).

**Figure 4:**
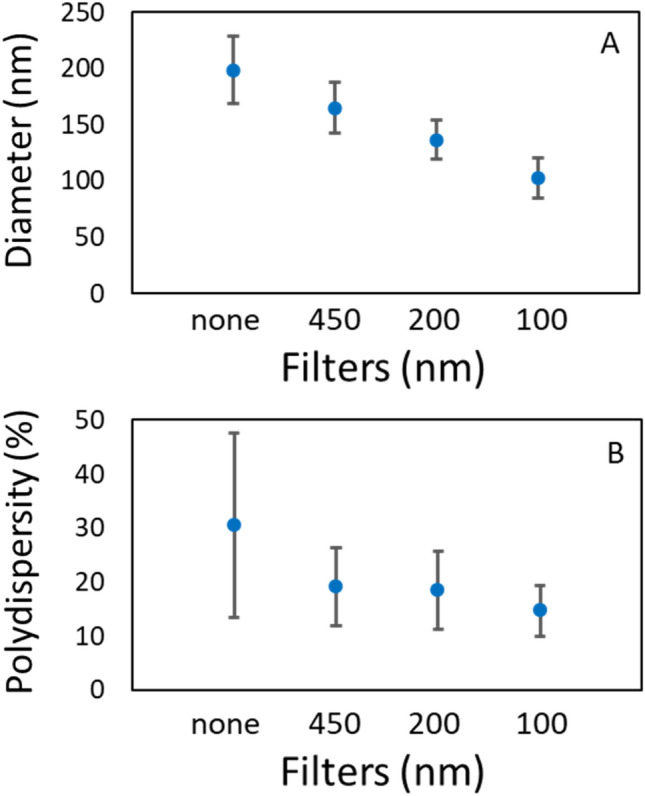
DLS measurements of filtrated PEVF. (**A**) The mean size and standard deviation for different filtrate of a 10× concentrated PEVF. Note that PEVF during the preparation was filtered through a 0.8 µm filter. (**B**) The corresponding polydispersity with standard deviation.

DLS can potentially detect smaller nanoplastics than NTA, however, no high-quality data could be recorded on the crude PEBD breakdown fractions. This could be due to either low particle concentration or due to severe polydispersity as particles of many sizes are present. To enhance the data quality and to determine if smaller PE nanoplastics is present, another batch of PEBD was made. The PEVF fraction was concentrated 10 times and subjected to filtration through 450 nm, 200 nm, and 100 nm filters. DLS measurements clearly shows that for each filtration step the size decrease together with the polydispersity index (Fig. 4). After the last filtration, the mean diameter is 103 nm, which is the size determined by NTA (Fig. 2). The observed size after three rounds of concentration steps is lower than for the PEBD starting material. The particle concentration is only marginally changed by the concentration steps. The concentration and cleaning of the PEVF fraction allowed for determining the apparent zeta potential for the particles in PEVF which is − 10.9 mV with a standard deviation of 6.4 (Fig. S2).

**Figure 5:**
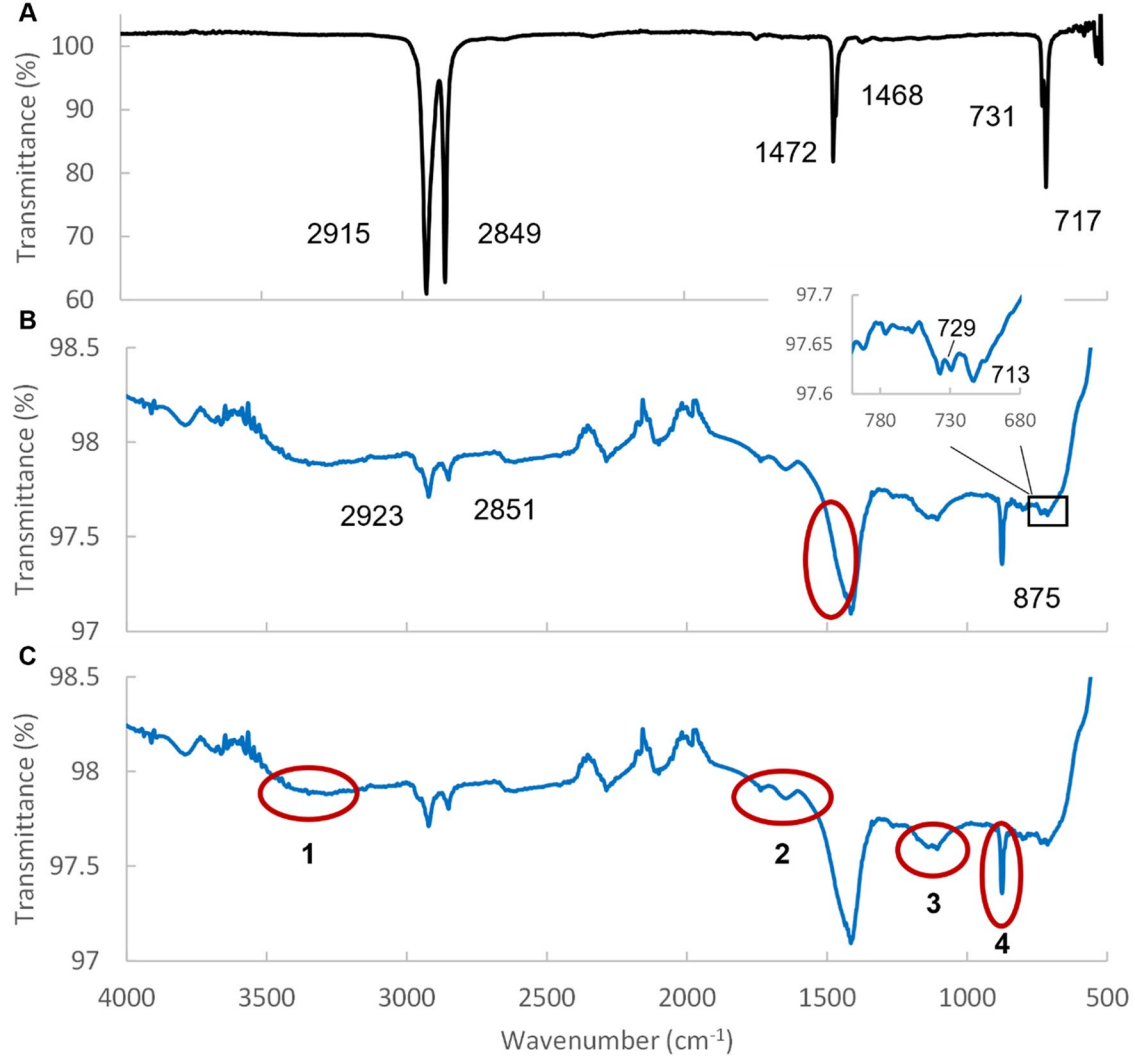
ATR-FTIR spectra of intact HDPE and PEVF. Spectra from the HDPE bag that was used as starting material for the breakdown process (**A**). Spectra from 10 times concentrated PEVF with the signature minima for either HDPE (**B**) or indicative for oxidation (**C**) marked in red circles.

The chemical signature of the HDPE bag that was used as starting material for the breakdown was determined by ATR-FTIR (Fig. 5A). The ATR-FTIR transmittance spectrum contains all the expected minima for HDPE which confirms that the bag is made of HDPE^29^. The spectrum is used as relevant reference spectrum in determining chemical changes during the mechanical breakdown. To obtain a high-quality spectrum from the breakdown products we used the cleaned and 10 times concentrated PEVF fraction (Fig. 5B). The spectrum differs from the spectrum from the HDPE bag, but the signature minima can still be identified. The area where minima 1468 and 1472 is obtruded but there is a shoulder in the peak in the region containing the two minima. Furthermore, the peak is relatively much stronger compared to the starting material, which indicates oxidation of the carbon chain^30^. There are several regions in the spectrum in which new signals can be detected. Some of these are indicated as 1 to 4 in Fig. 5C. Region 1 is probably due to formed hydroxyl, region 2 to carbonyl groups and region 3 to carbon-oxide bonds^31^. The sharp minima at 875 cm^−1^ in region 4, is unusual but can indicate the formation of carbonate ions, CO_3_^−2^^32^. Overall, the ATR-FTIR transmittance spectrum of the PEVF strongly indicate that we have highly oxidated HDPE in the fraction. The spectra obtained from PEBD and FT fractions are of lower quality (Fig. S3) but likewise indicate that these fractions, including the FT fraction, contain oxidised HDPE. This suggests that there are short HDPE chains in the FT fractions and thereby also in PEBD. Calcium carbonate is sometimes used as an additive in HDPE^33^ which could explain the minimum at 875 cm^−1^. However, the minimum is only present after breakdown but not in the starting material, which may indicate that the minimum is due to strong oxidation rather than additives.

Overall, the characterization of the products from the breakdown procedure shows that nanoplastics are formed from HDPE with sizes between 80 and 300 nm with a peak at around 110 nm (it may be lower but due to the detection limit of NTA we do not know if there are formed particles under 80 nm). Compared to the much-used commercial polystyrene nanoparticles the size range of PE breakdown particles is broad, the carbons are differently oxidized and the morphology is diverse. The crude breakdown product can be divided, by size fractionation, to PEVF, highly oxidized HDPE nanoplastics in the same size range, and FT fraction with no measurable nanoplastics but that likely contain short chains of highly oxidized HDPE and possibly additives released from the plastic during the breakdown process.

### Long term toxicity of the HDPE breakdown fractions

#### Toxicity test 1 on *D. magna*: high concentration of flow through molecules

The PEBD, PEVF, and FT were tested for toxicity towards *D. magna* in life-time experiments. Before the experiment the PEVF was diluted five times to attain the same concentration as in PEBD. This mean that the nanoplastics concentrations are the same in PEBD and PEVF. The concentration of FT in the samples were 100% in PEBD, ~ 20% in PEVF, and ~ 100% in FT. The different fractions used in the toxicity test is described in Table 2. The prepared HDPE particles are not stable in solution over time, no nanoparticles could be detected by NTA in a sample that had been standing on the lab bench for 100 days (Fig. S4). We have not been able to measure the particle size after addition of algae.

**Table 2:** The different fractions from PE breakdown and in which toxicity test they are used.

Fractions	Contains	Used in test	
		1	2
PEBD	Breakdown PE after filtration through 0.8 µm filter	X	
FT	Small molecules after filtration of PEBD through a 10 kDa filter	X	
PEVF	PE nanoplastics and approximately 20% FT	X	
3rd PEVF	PE nanoplastics in approximately 2 times concentration compared to PEVF and less than 1% FT		X
3rd FT	Less than 1% FT		X
Control	Tap water	X	X

**Figure 6:**
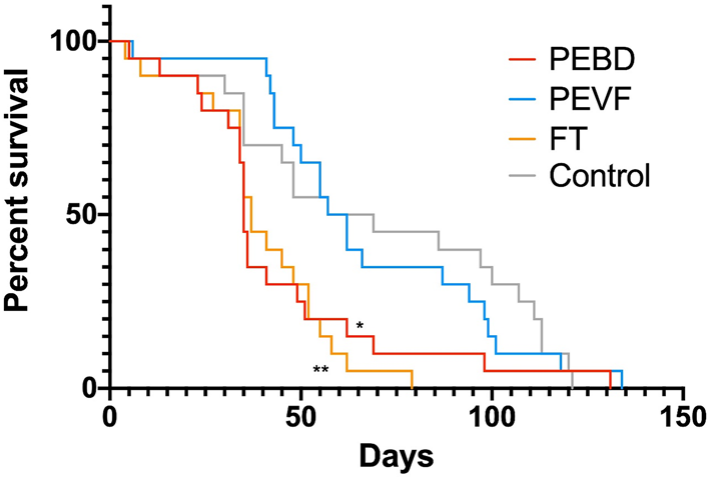
The survival of *D. magna* after exposure to one initial dose of the HDPE breakdown fractions. *D. magna* was exposed to a single dose of the different breakdown fractions. The PEVF contains, in addition to nano-sized plastics, approximately 20% FT. Asterisks indicate significant difference from a control group, *p < 0.05, **p < 0.01.

The mortality is significantly higher when *D. magna* is exposed to PEBD, and FT compared to the control (Fig. 6, Table 3). However, there is no significant difference in lethality between PEVF and a control group (Fig. 6, Table 3). Similarly, there is no significant difference between PEBD and FT. Consequently, at these concentrations and experimental conditions HDPE nanoplastics, fraction PEVF, is not toxic to *D. magna*, whereas the FT is. Furthermore, the presence of HDPE nanoplastics to the FT, fraction PEBD, do not increase the toxicity.

**Table 3:** Statistical analyses of the life-time toxicity test.

	Mantel–Cox-test		Geham–Breslow–Wilcox-test	
	χ^2^_(df=1)_ =	p value	χ^2^_(df=1)_ =	p value
Control vs. FT	10.06	**	5.617	*
Control vs. PEBD	3.279	n.s.	4.477	*
Control vs. PEVF	0.08191	n.s.	0.001644	n.s.
FT vs. PEVF	13.06	***	11.69	***
FT vs. PEBD	0.1052	n.s.	0.06593	n.s.
PEVF vs. PEBD	6.162	*	10.39	**

Significant difference represented in the form of asterisks and p-value between the test groups. *df* denotes degrees of freedom, *n.s*. denotes no significant difference.

*p < 0.05, **p < 0.01, ***p < 0.001.

The Mantel–Cox test (also known as the log-rank test) is the most commonly used. The Geham–Breslow–Wilcox method can sometimes be better suited as it gives more weight to early deaths and does not require the hazard to be the same trough out the test period. In this experiment it could be that the toxicity is higher in the beginning when the particles are added, it can also be argued that the *D*. *magna* are more sensitive at an early life stage and that their deaths should be weighed less in the beginning. The results of both tests are therefore reported to get the most comprehensive understanding of the results.

**Figure 7:**
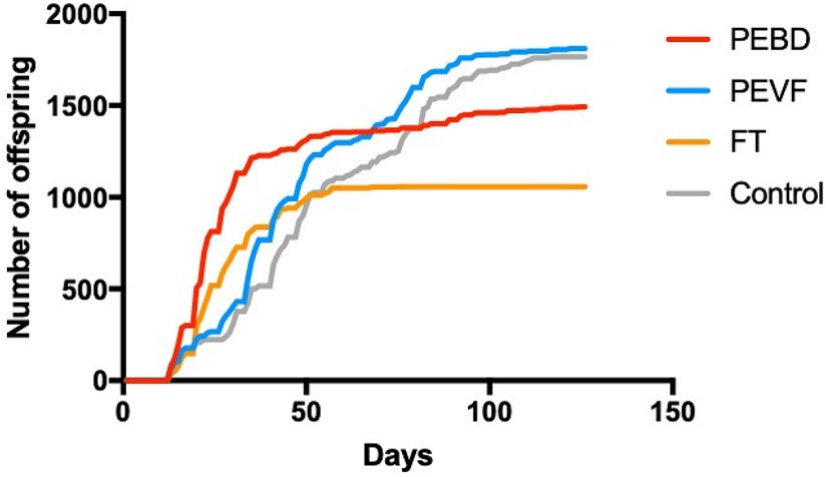
Cumulative number of offspring for each test group. *D. magna* were exposed to a single dose of the different breakdown fractions. The PEVF contains, in addition to nano-sized plastics, approximately 20% FT.

The rate of reproduction is somewhat higher for PEBD and slightly higher for FT in the beginning (Fig. 7). This can possibly be explained by stress caused by the particles or additives. Analysing the whole experimental period revealed no difference between PEVF nor PEBD in comparison to the control (Tukey’s multiple comparison p always > 0.05). However, the rate of reproduction for FT was significantly lower in comparison to the control, PEBD and PEVF (Tukey’s multiple comparison p always < 0.05). The reproduction was further analysed as number of offspring per animal and day lived using a one-way ANOVA and Tukey’s multiple comparison test that compares the mean of every group (Fig. S5). This reveals a significant difference between PEBD and control only (p < 0.05), which indicates that that *D. magna* individuals are stressed also in PEBD.

##### Toxicity test 2 on *D. magna*: high concentration of PEVF and low concentration of FT

**Figure 8:**
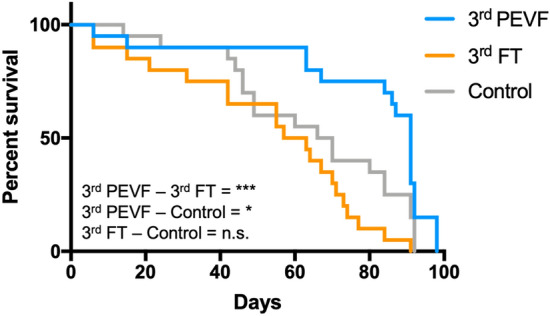
The survival of *D. magna* when exposed to weekly doses of the HDPE breakdown fractions. The PEVF is concentrated two times compared to toxicity test 1, and FT concentration is below 1% compared to *D. magna* test 1. Asterisks indicate significant difference from a control group, *p < 0.05, ***p < 0.001.

The lack of or very low toxicity of PEVF was confirmed in a second life-time toxicity test. Before the test, the PEVF was purified from FT and concentrated to twice the concentration in the previous experiment and is called 3rd PEVF. The FT fraction used in this second life-time experiment is from the last purification step, i.e., the concentration is about 1%, and called 3rd FT. To further increase the exposure, the *D. magna* were exposed to new 3rd PEVF or 3rd FT every week. Each new preparation was characterized for size. Compared to the control no increased lethality could be seen for 3rd FT (Mantel–Cox: χ^2^_(1)_ = 3.405, p > 0.05; Geham–Breslow–Wilcox: χ^2^_(1)_ = 1.838, p > 0.05) (Fig. 8). In contrast, there is decreased lethality for 3rd PEVF compared to both control (Mantel–Cox: χ^2^_(1)_ = 5.753, p < 0.05; Geham–Breslow–Wilcox:, χ^2^_(1)_ = 5.46, p < 0.05) and 3rd FT (Mantel–Cox: χ^2^_(1)_ = 18.39, p < 0.001; Geham–Breslow–Wilcox:, χ^2^_(1)_ = 13.73, p < 0.001), possibly due to bacterial growth on the 3rd PEVF particles that would be an additional food source for *D. magna*. The number of offspring increased for 3rd PEVF fractions late in the study. However, the number of offspring per lived day showed no significant difference in comparison with a control group (p > 0.05).

Mechanical breakdown of HDPE produces nanoplastics of highly oxidized PE at sizes between 80 and 300 nm with a peak around 100 nm. There is no evidence of smaller nanoplastics in particle form. However, the breakdown products can be separated into a nanoplastics fraction and a fraction with smaller molecules with a chemical signature of highly oxidized HDPE. The purified PE nanoplastics fraction is not toxic to *D. magna* at these concentrations but it should be noted the concentrations used are still much lower than the concentrations of model PS nanoparticles showed to be toxic in similar life-time tests^34^ or in experiments recording sublethal effects on *D. magna*^35,36^. However, interestingly breakdown media containing smaller molecules that are toxic to *D. magna*. This result emphasizes the importance of careful isolation and characterization of the nanoplastics before evaluation of its biological effects can be done. Similar results were reported for leached material from teabags as the microplastics had less impact than the starting material^14^. However, the effect of the smaller molecules was not tested.

One possible source of small molecules is the leaching or generation of short PE-chains (oligomers). The oxidized oligomers are probably not toxic, and oxidation of the oligomers has for example been shown to enhance the biodegradability by a Gram-positive bacteria *Rhodococcus rhodochrous*^37^. Another source of small molecules are additives that are used to give HDPE desired properties. These include antioxidants, flame retardants, plasticisers, heat stabilizers and many more. The exact composition of these additives is usually only known by the manufacturer. Additives, monomers, and oligomers can leach out from the plastic and into the surrounding water. Previous studies performed on leaching from plastic can give us some insight. A screening study on *D. magna* on leachates from plastic^38^ tested 32 different plastic products under two different leaching methods. This study showed that there was no toxicity observed for *Daphnia* from the HDPE products tried, such as sand shovel and drinking water pipes. On the other hand, another study^39^ showed a toxicity for one (watering can) out of five tested HDPE product leachates. They suggest that the main cause of the toxicity was the hydrophobic organic molecules. However, these studies focused only on leaching and not mechanical breakdown. We believe it can be assumed that the mechanical breakdown will generate a larger concentration of both oligomers and of leachates because of the increased surface area.

The mechanical breakdown of HDPE results in irregularly shaped particles with surprising levels of oxidation in the nanoplastic fraction. The irregularity is especially interesting as irregular shaped PE microplastic have a more adverse effect on *D. magna* than spherical particles^25^. In nature, these particles will be submitted to UV irradiation which have resulted in oxidation of macro- and micro-plastic^21^. For future studies it would be interesting to evaluate how UV irradiation will affect the size and morphology as well as toxicity of the already highly oxidized nanoplastics and FT fractions.

In this article we have shown that mechanical wear can produce HDPE nanoparticles from bulk HDPE. The produced HDPE nanoparticle fraction is toxic to *D. magna* in a long-term toxicity test. However, it is not the HDPE nanoparticles that cause the toxicity. Instead, it is the small molecules: chains of PE that has been broken from the particles, and potential solved additives that cause the toxicity. These results add to the understanding on how breakdown plastic can act as a toxic pollutant towards organisms. Furthermore, it adds crucial information on what fractions/components of breakdown plastic that can be a potential future environmental problem.

## Supplementary Information

Supplementary Information.
