# Supplementary material for: Size fractionation of high-density polyethylene breakdown nanoplastics reveals different toxic response in Daphnia magna
Source: Sci Rep. 2022 Feb 24;12:3109. doi: 10.1038/s41598-022-06991-1 (PMC8873248; doi:10.1038/s41598-022-06991-1)
Supplement: Supplementary file 1 — Supplementary Information. [file 41598_2022_6991_MOESM1_ESM.docx]

# Supplementary Information

Size fractionation of high-density polyethylene breakdown nanoplastics reveals different toxic response in *Daphnia magna*

Mikael T. Ekvall^1,2^, Isabella Gimskog^2,3^, Jing Hua^2,3^, Egle Kelpsiene^2,3^, Martin Lundqvist^2,3^, and Tommy Cedervall^2,3*^

^1^Aquatic Ecology Unit, Department of Biology, Ecology Building, Lund University, SE-223 62 Lund, Sweden

^2^NanoLund, Lund University, Box 118, SE-221 00 Lund, Sweden

^3^Biochemistry and Structural Biology, Lund University, Box 124, 22100 Lund, Sweden

*Correspondent author: e-mail: tommy.cedervall@biochemistry.lu.se

**Table S1: Tap water used in the toxicity studies.** Information about the tap water in Lund from the local water supplier (VA Syd), the valued are averaged from 350 tests taken in the time-period April 2020 to September 2020. https://www.vasyd.se/Artiklar/Dricksvatten/Kvalitetskontroll-av-dricksvatten

| pH | PO4  (mg/L) | NO3  (mg/L) | Cu  (mg/L) | Cl  (mg/L) | Fe  (mg/L) | Microorganisms  (cfu/mL after 3 days at 22°C) | Turbidity  (FNU) |
| --- | --- | --- | --- | --- | --- | --- | --- |
| 8.4 | 0.008 | 1.4 | <0.05 | 29 | 0.02 | 8 | 0.2 |


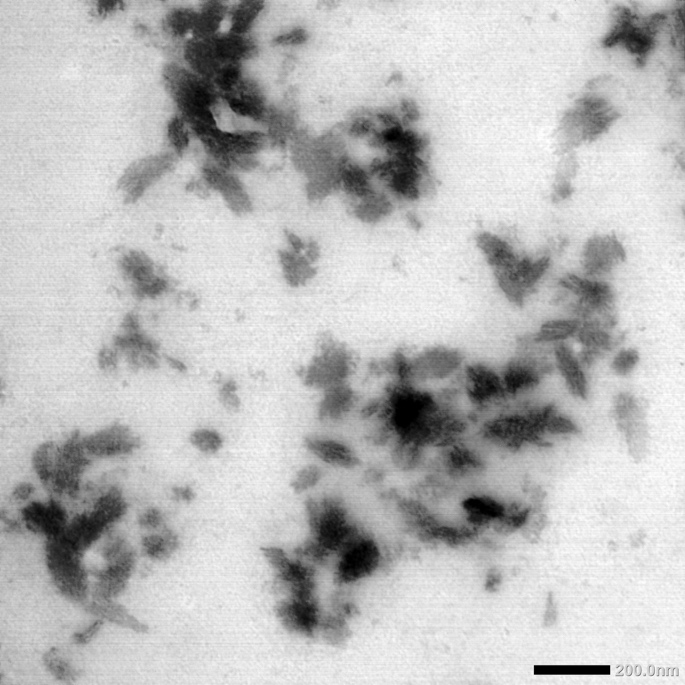

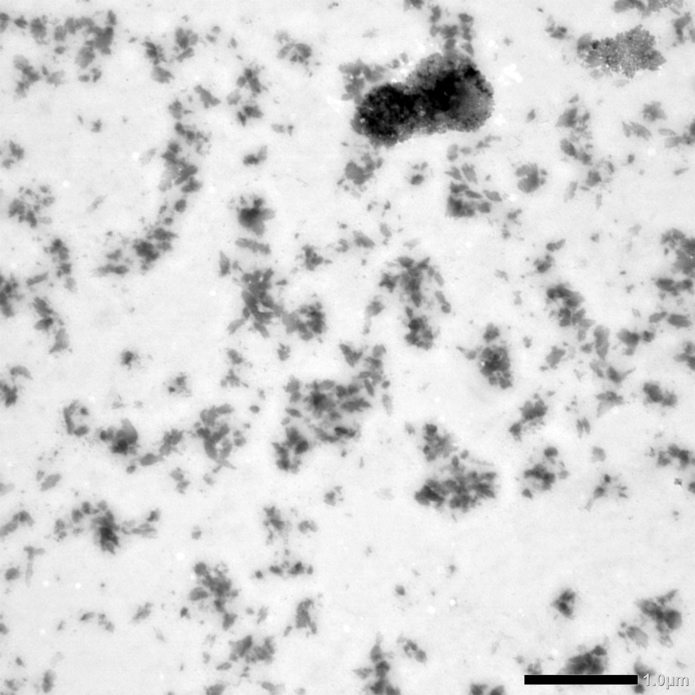


**Figure S1**. **TEM-images of brake-down HDPE**. To the left 6000 times magnification and a scale bar of 1.0 um and to the right 20k times magnification and a scale bar of 200 nm.

**Figure S2 Zeta potential for the concentrated PEVF samples.** Dark red: after concentration step 1, orange: after concentration step 2 and blue: after concentration step 3. Three repeats of each sample.


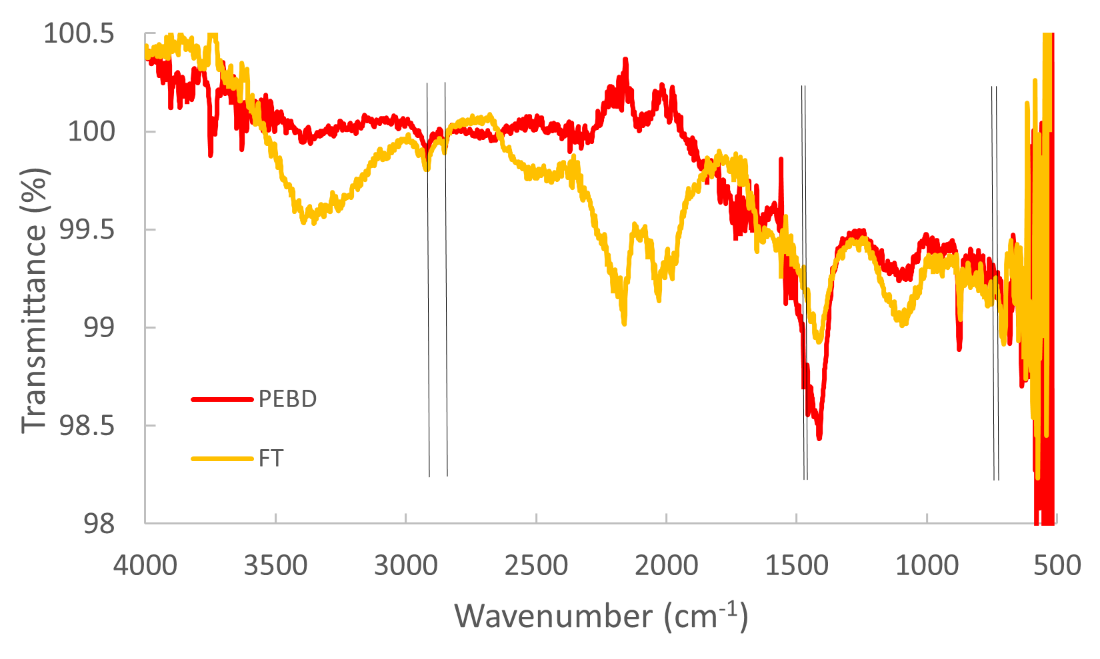


**Figure S3. ATR-FTIR spectra of different HDPE breakdown fractions.** The grey vertical lines indicate where the peaks of a pure HDPE spectra should be.


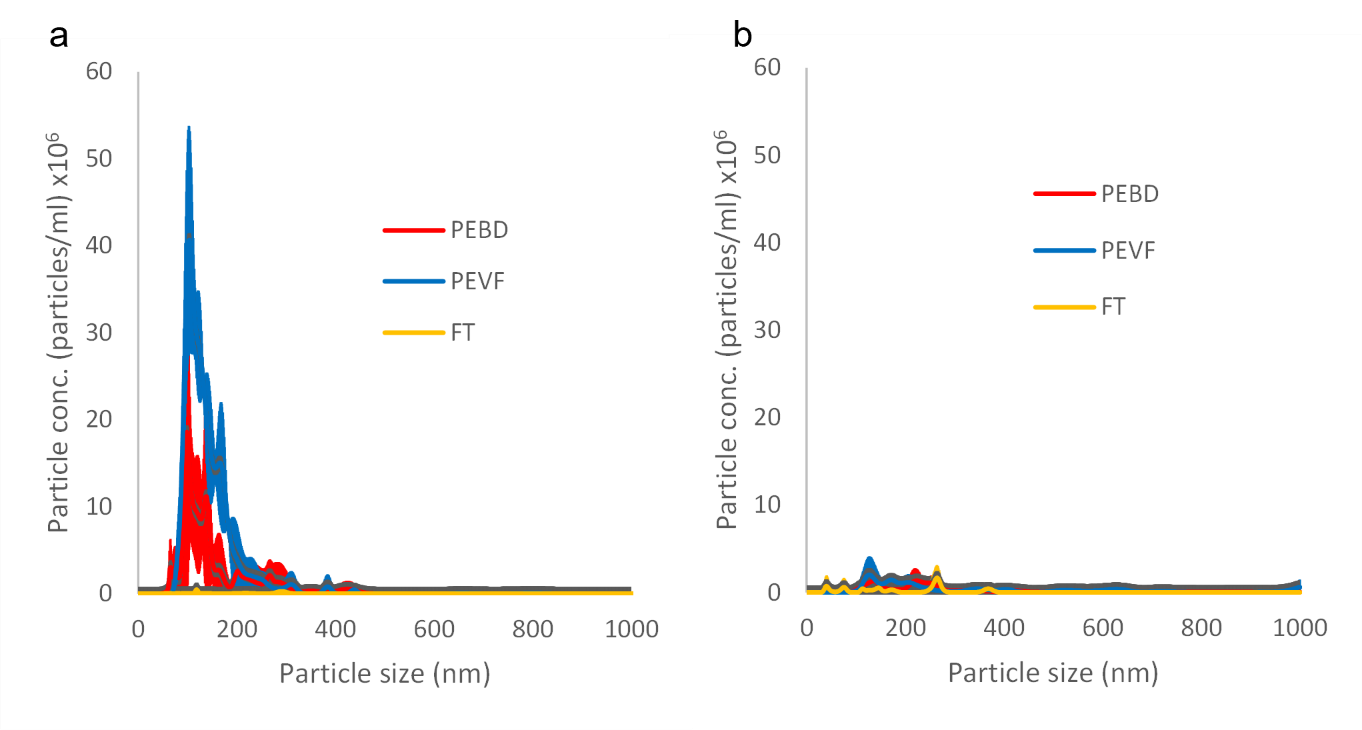


**Figure S4. NTA from start (to the left) and after 100 days (to the right).** All NTA data showen is averaged from three runs.


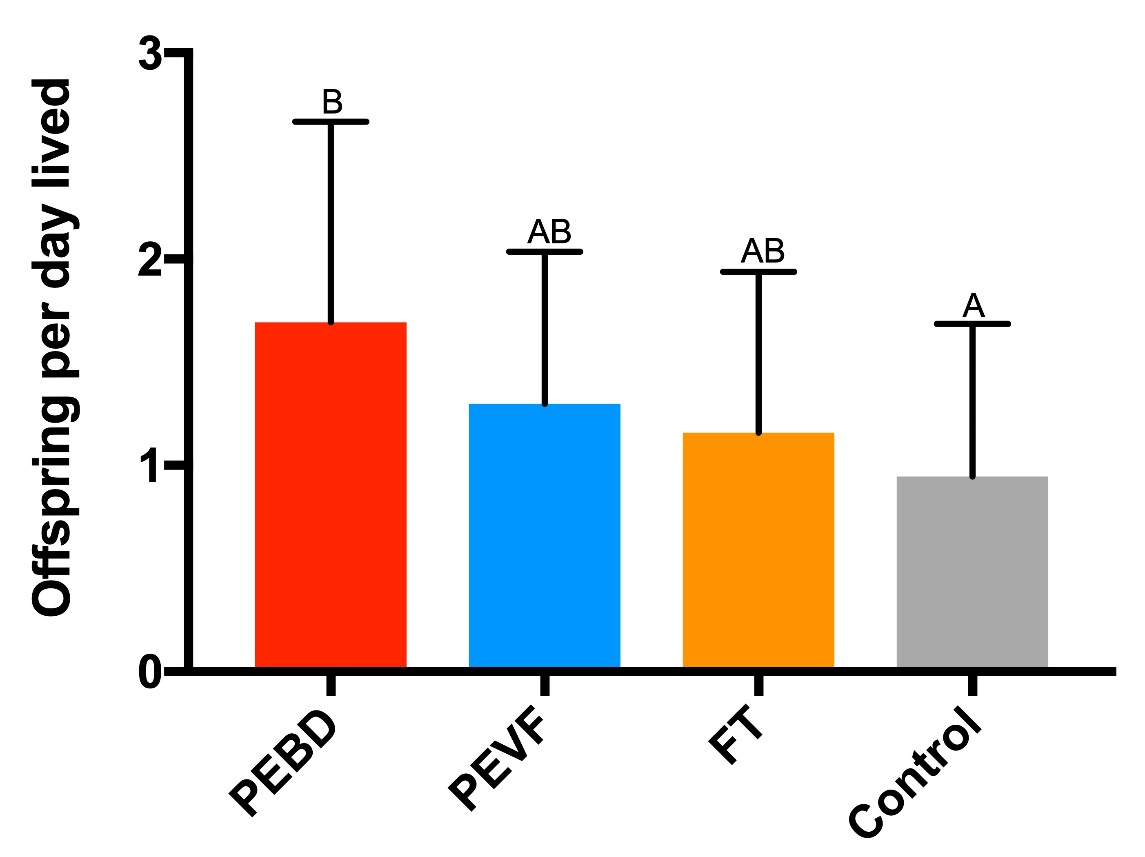


**Figure S5. Reproduction data from D. magna test 1.** Results of one-way ANOVA and Tukey’s multiple comparison test for the reproduction of the groups, the test is calculated as number of offspring per individual and day alive. Different letters indicate that PEBD is significantly different from the control.
